# MCGC-Net: A Text-Enhanced Geometry-Consistent Network for UAV-Based Road Crack Detection

**DOI:** 10.3390/s26113487

**Published:** 2026-06-01

**Authors:** Zhoujun Ou, Shicong He, Rongwei Bu, Peng Wang, Gufeng Gong

**Affiliations:** 1 School of Transportation, Changsha University of Science and Technology, Changsha 410114, China; ouzhoujun@163.com; 2 School of Electronics, Information and Physics, Central South University of Forestry and Technology, Changsha 410004, China; heshicong88@gmail.com (S.H.); t20252936@csuft.edu.cn (P.W.); t20090523@csuft.edu.cn (G.G.)

**Keywords:** UAV remote sensing, road crack detection, multimodal fusion, image-text joint modeling, slender crack localization, intelligent road inspection

## Abstract

**Highlights:**

**What are the main findings?**

- MCGC-Net integrates text semantics and geometry-aware crack modeling.
- The proposed framework improves crack detection in complex UAV scenes.

**What are the implications of the main findings?**

- Semantic guidance reduces confusion from shadows and pavement textures.
- Geometry-consistent learning improves localization of slender cracks.

**Abstract:**

With the rapid development of unmanned aerial vehicle (UAV) remote sensing and deep learning, road crack detection has become an important component of road condition assessment and intelligent road maintenance. However, accurately detecting cracks from UAV images remains challenging due to complex background environments, slender crack structures, blurred boundaries, and irregular crack shapes and orientations. Traditional methods that rely solely on visual information often struggle to achieve stable and accurate detection performance under these conditions. To address these challenges, this paper proposes a Multimodal Crack Geometry-Consistent Network (MCGC-Net) for high-precision road crack detection in complex road scenes. First, a UAV-based multimodal road crack dataset with image-text annotations is constructed. Specifically, crack-related textual descriptions are automatically generated from crack annotations using predefined semantic templates, which summarize crack morphology, spatial distribution characteristics, and structural properties. These semantic descriptions provide high-level semantic prior information for crack representation learning. Second, a Multimodal Contrastive Semantic Gating module (MCSG) is introduced to leverage automatically generated crack semantic descriptions and in-batch image-text semantic differences to guide visual feature learning, thereby improving the discrimination between crack and non-crack regions under complex background conditions. Furthermore, a Crack-Aware Slenderness Loss (CASL) is proposed to explicitly constrain slenderness consistency between predicted boxes and ground-truth boxes, improving localization stability for slender crack targets. In addition, a KAN-based Nonlinear Channel Attention mechanism (KAN-CA) is introduced to enhance feature representation capability for complex crack structures. Experimental results demonstrate that the proposed MCGC-Net effectively improves crack detection accuracy and structural representation capability under complex road environments. The proposed method provides a practical and reliable solution for UAV-based intelligent road crack detection.

## 1. Introduction

Road cracks are one of the earliest and most widespread types of roadway wear; it often occurs on high-speed expressways, city roads, etc. [1], due to heavy vehicular traffic load, frequent freezing–thawing cycles from weather condition changes, and environmental factors’ impact. Cracks will allow moisture and foreign objects to infiltrate inside the pavement layer when they occur; it is expected that this will result in a decrease in the bearing capacity of the underlying base material, leading to further deterioration of the road structure at an accelerated pace. Moreover, repeated exposure to environmental factors over time may exacerbate damage, such as potholes or sinking. These problems increase maintenance costs and shorten the service life of the road. At the same time, continued crack growth may create risks for driving safety and bring more pressure to road operation and management. For these reasons, accurate identification and detection of road cracks are very important for early diagnosis, proper maintenance, and traffic safety.

Traditionally, the road crack situation is observed by people. Inspectors inspect the road surface on-site to determine its position and the shape of cracks. This will directly show how good or bad it has changed in recent days. However, due to practical difficulties such as limited manpower, time required for collection, etc., it cannot be carried out frequently at present. To improve detection efficiency, researchers later developed automatic crack detection methods based on image processing [2]. These methods depend on the differences in gray levels and shapes or sizes of objects in the image. They perform cracking detection by applying threshold division, edge extraction, and morphological transformation processes to obtain visualized results but it may not be suitable for applications where there is too much clutter, such as shadows or oil stains, due to rain. Due to this reason, fixed rule-based image-processing techniques are prone to fluctuations in their parameters and changes in the scene; thus, they cannot maintain consistent recognitive accuracy over a long period. Thereafter, several scholars began exploring the application of machine learning technology in cracking recognition studies, most of which started their study through building a hand-made feature space based on texture information (such as edge direction), gradient value, etc., and then classified samples belonging to cracks or not according to this new representation after the classifier was trained. In comparison to rule-driven methods, it is relatively robust due to the flexibility provided for parameter adjustment. Nevertheless, whether it is effective will largely be influenced by problems like selecting features or processing data during preprocessing. A deep neural network (DNN) works differently. By directly extracting hierarchical features in the learning process through end-to-end training, reducing dependence on pre-designed features, it is also beneficial for strong feature representation and better generalization in complex road environments. For these reasons, deep learning has been widely used in this field, and it has achieved better precision and more robustness under perturbation.

For example, Cha et al. proposed a crack detection method based on convolutional neural networks (CNNs) [3]. Using a sliding-window approach, it classified the image patches and detected cracks automatically. It is one of the early applications of deep learning in identifying concrete defects that surpasses traditional approaches in terms of feature representation because it can learn directly from raw data. However, it requires local window-based classification. Therefore, the method was unable to fully utilize the global context information. It still had a tendency for false alarms in complicated background environments and texture noise. Maeda et al. released the large-scale Road Damage Dataset [4]. Furthermore, using a one-stage detection model like SSD or YOLO to detect various kinds of road damage simultaneously is adopted here. Their work has made crack detection capable of working in real time. However, a fixed-anchor mechanism decreased its matching ability for small and thin cracks. Therefore, the detection accuracy is still unsatisfactory. Fan et al. proposed a crack detection method that combined deep convolutional neural networks with adaptive thresholding [5]. This approach first obtained deep features of the images and then added post-processing to make the cracks appear smoother, continuing to use the threshold function for subsequent operations. Therefore, there is an obstacle to its full-range recognition. In terms of robustness, its performance remained poor under various road conditions and complicated pavement types. Li et al. introduced multi-scale feature fusion and attention mechanisms into an improved YOLO framework [6]. This approach has raised the crack detection rate for different scales, performing relatively well with mid-size cracks. However, it cannot effectively show very small or scattered fine cracks. Xu et al. developed a crack instance detection method based on Mask R-CNN [7]. Their approach proposed a region proposal network for precise location, which has obtained excellent performance at the level of instances. However, cracks frequently exhibit irregular shapes. Due to these reasons, the algorithm still failed to detect some objects correctly in complex backgrounds or with low contrast. Although there have been advancements in object-detection-based techniques for detecting road cracks, it is not yet perfect.

**Figure 1:**
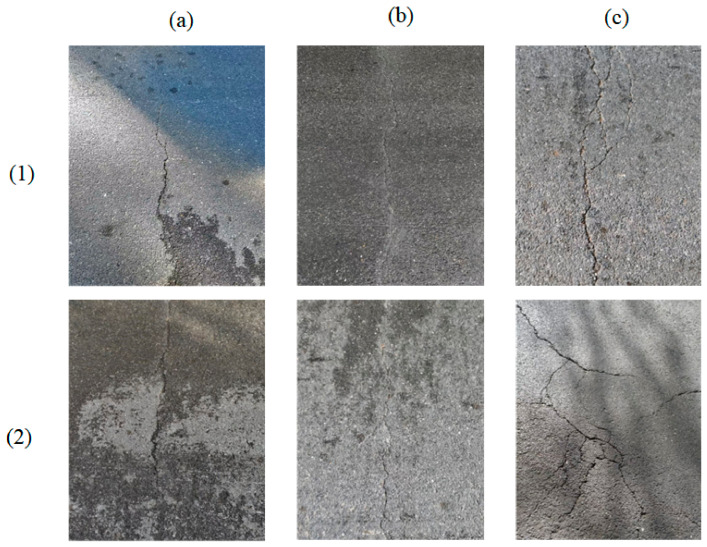
Facing challenges. (**a**) Complex background interference caused by shadows, oil stains, water marks, and wheel tracks with crack-like visual characteristics; (**b**) Small-width cracks with blurred boundaries and low contrast, making accurate crack recognition and localization difficult; (**c**) Slender, discontinuous, and irregular crack structures, which increase the difficulty of long-range structural modeling.

(1) As shown in Figure 1a, real-world road images usually have shadows, oil stains, wheel tracks, water marks, etc., which are all complex textures and backgrounds that are relatively close in color to cracks; therefore, the model will be more difficult to distinguish between them. This problem will lead to incorrect recognition and deteriorated bounding box performance. It may reduce the dependability of its final results’ identification. Examples of automatically generated textual descriptions are as follows: (a1) A slender longitudinal crack extends across the pavement surface under shadow interference and uneven illumination conditions. Water stains and pavement contamination reduce the local contrast between the crack and surrounding regions, making the crack boundary difficult to distinguish from the complex background texture. (a2): A longitudinal crack appears on a severely worn pavement surface with irregular local morphology. Complex pavement textures, brightness variations, and degraded surface regions lead to discontinuous crack boundaries and unstable structural responses.

(2) Figure 1b shows that the width of some road cracks is quite small, with unclear edges. These cracks are also often like that of the surrounding pavement surface. More severe in early-stage cracks or severely worn pavements, the crack outlines are hard to identify due to their complexity. In most cases, the gray scale differences between cracks and backgrounds are relatively small. Therefore, this kind of problem may result in an inability to accurately locate or a missed detection of very small cracks. Also, the model’s performance may decline during training. Examples of automatically generated textual descriptions are as follows: (b1): A weak and slender crack is distributed in a low-contrast pavement region where the crack appearance is highly similar to the surrounding gray surface texture. Blurred boundaries and coarse background patterns interfere with the extraction of fine crack structures. (b2): Small and discontinuous crack fragments are distributed within a complex textured pavement background. Due to the narrow crack width, weak grayscale contrast, and surrounding texture interference, the crack structures are difficult to perceive and accurately identify.

(3) Figure 1c shows that cracks frequently occur in thin and long parts. These cracks are also often discontinuous, with uneven directions, and may extend over a wide range in one image; traditional convolutional networks primarily utilize small local receptive fields to identify features because they cannot capture long-distance structural patterns of cracks easily; therefore, such problems may lead to misjudgment or an incomplete location in some dislocated-crack areas. This might impact the general structure and uniformity of the detection result. Examples of automatically generated textual descriptions are as follows: (c1): A relatively long crack extends longitudinally across the pavement with irregular curvature and several local branching structures. The crack width changes along the extension direction, resulting in unstable continuity and increased structural complexity. (c2): Multiple intersecting and branching cracks form an irregular crack network under striped shadow interference. Significant directional variations, discontinuous structures, and uneven illumination conditions increase the difficulty of capturing long-range crack patterns.

To enhance the crack detection effect of complex road scenes, recently, more research has been conducted along these paths: reorganizing detectors, enhancing attention mechanisms and establishing Global context models. Hu et al. proposed an improved YOLOv5-based method for road crack detection [8]. Their targeted image type mainly included vehicle–mountain-road type, low-resolution blurry small-crack, complex background and incompletely recorded crack situations. Moreover, they also added the slim-neck structure to enhance crack feature representation and improved their own system’s detection accuracy in difficult situations through this design. Lan et al. introduced an attention mechanism into a YOLOv5-based crack detection framework [9]. Compared with YOLOv3, YOLOv5s, and YOLOv5s-attention, respectively, it was found in their studies that attention-enhanced models have stronger adaptability to important crack locations. Guo et al. proposed a Transformer-based pavement crack detection network [10]. They experimentally verified that the designed scheme is effective against interference and can find crack images under various conditions efficiently. Zhang et al. further applied Transformer-based segmentation models, including TransUNet, SwinUNet, and MTUNet, to pavement crack detection [11]. Using data sets containing oil stains, vegetation and shadow interference, they found through experiments that applying a deep contextual model to recognize cracks in such environments is feasible. The improvement of this method includes enhancing visual features, constructing attention models and extracting global context information. However, the above approaches are still primarily based on local/global texture, edge information and visual response for making detection decisions. Due to this reason, these methods do not have direct access to high-level semantic information; when it comes to road backgrounds with shadows, oil spots, wheel scuffs, etc., that resemble cracks in detail, these models will still generate false alarms and inaccurate boxes.

Recent research has focused less on describing the fine crack structures through super-resolution reconstruction, multi-scale feature fusion and other methods for improving localization accuracy. Xiang et al. studied micro-crack recognition in UAV crack images with low resolution and motion blur [12]. A new approach of automatic micro-crack recognition based on high-resolution reconstruction and semantic segmentation was proposed, experimentally showing that improved detail re-gain could enhance the detection accuracy of tiny cracks. Ye et al. further improved an encoder–decoder model [13]. By using multi-scale feature fusion, they improved the pixel-level crack detection accuracy for infrastructure images acquired under night conditions. The results show that richly detailed information at various scales can help alleviate problems such as blurred crack boundaries due to blur. Niu et al. proposed a method that combines dynamic snake convolution with an attention mechanism [14]. This approach enhances the representation of elongated and irregular crack structures and improves the modeling capability of complex crack patterns. Although these studies have improved the detection of road cracks to a certain extent, most of them are primarily based on enhancing features or supporting image segmentation when dealing with small crack patterns. They cannot represent the slender geometrical priors of cracks explicitly at present. As a result, there may be inaccuracies in localization, deformed bounding boxes, or missing detection boxes for small cracks in scenes containing target objects with smaller sizes, unclear boundary conditions, and weaker texture effects. In short, at present, these algorithms are still insufficient in capturing subtle geometric details.

To further reveal the fine and long crack structures, some studies are now focusing on global-context-based modeling, long-distance-dependency-learning-based methods, etc., to enhance the structural continuity of crack identification. Xu et al. proposed a locally enhanced Transformer network called LETNet [15] for high-resolution pavement crack detection. Their results demonstrated that enhancing long-range contextual modeling through attention mechanisms could effectively improve crack detection performance. Xiao et al. further proposed CrackFormer [16]. Hybrid Window Attention and the proposed high-resolution transformer together integrate multiple resolutions’ semantic information to construct an improved model. Their results showed that stronger long-range dependency modeling across different spatial locations can improve the continuity of thin cracks and complex crack patterns. Wang et al. proposed a global graph multi-scale network called GGMNet [17]. Their model improves pixel-level pavement crack detection through global graph modeling and multi-scale feature interaction. This design helps the model better represent complex crack structures and the overall shape of cracks. Ashraf et al. studied road pavement crack detection, classification, and segmentation by combining multi-scale feature aggregation with Transformer-based attention mechanisms [18]. Their results showed that global semantic information and multi-scale structural cues can jointly improve the recognition of crack targets with different shape features. Although these studies have improved road crack detection to some extent, most of them still mainly use traditional attention mechanisms or linear mapping methods for feature enhancement. These methods still do not directly capture the complex nonlinear structural changes in cracks. Because of this, current methods still have obvious limits when they deal with cracks that are thin, broken, and irregular in direction. These methods are not suitable for describing the overall structural continuity and consistency of cracks in this way.

To solve the above problems, this paper proposes a multimodal crack geometry-consistent network (MCGC-Net) to detect cracks on roads; it integrates automatically generated crack semantic descriptions derived from crack annotations into the detection framework to provide semantic guidance under complex background conditions and uses a multilingual contrastive semantically gated module to help the model pay more attention to the semantic characteristics related to cracks, and thus improve its ability to distinguish between actual crack areas and ambiguous background information. In addition, this paper proposes a crack-awakened slenderness loss function for improving the geometric-regression performance on detailed cracks. This loss will help the model find thin and small cracks more precisely. The model also adds a nonlinear channel attention mechanism based on the Kolmogorov–Arnold Network to help the model better identify crack structure components such as thin extensions, dispersed distributions, and various orientations. These designs improve detection accuracy via this proposal’s architecture. It can handle a more complex background, has higher accuracy under environmental noise, and obstacles less clear than blurring edges and rough crevices are available. The main contributions of this paper are as follows:

(1) This paper proposes a Multimodal Crack Geometry-Consistent Network (MCGC-Net) for UAV-based road crack detection. The proposed framework integrates visual information with automatically generated crack-related semantic descriptions derived from crack annotations. Specifically, crack morphology, spatial distribution, and structural characteristics are converted into textual semantic representations using predefined templates, providing semantic prior information for crack detection. The proposed multimodal framework further combines semantic guidance, geometric constraints, and structural representation learning to improve intelligent crack identification under diverse road conditions.
(2) The proposed Multimodal Contrastive Semantic Gating Module (MCSG) introduces automatically generated crack-related textual descriptions associated with UAV images as semantic guidance signals. These descriptions are generated from crack annotations and summarize crack attributes, including morphology, continuity, slenderness characteristics, and spatial distribution patterns. The module further introduces contrastive semantic gating based on semantic differences between image–text pairs within each batch, enhancing multimodal feature interaction and adaptive channel-response modulation. This design reduces feature ambiguity between crack and non-crack regions under challenging environments involving shadows, oil stains, wheel ruts, and complex pavement textures while introducing only limited additional computational overhead.
(3) A crack-aware slenderness loss function, named CASL. This loss directly models the aspect-ratio consistency between predicted boxes and ground-truth boxes. Adjust the loss weight according to changes in the slenderness of crack target points. Thus, the loss provides prior geometric information to be added during the regression step for thinning cracks. It helps reduce the instability of traditional IoU-based loss functions for small or thin crack targets compared with them. Therefore, to some extent, it improves the localization accuracy for small target detection that has blurred edges or insufficient texture features.
(4) This paper introduces the KAN-NonlinearChannelAttention mechanism based on KAN. It introduces KAN-based non-linear mapping to the channel attention mechanism. It strengthens the representation model of higher-order relations among channel features. It provides adaptive recalibration of the content related to cracks. Through these means, it can enhance the depiction of crack structures with long extensions, discontinuous distributions and irregular orientations. Therefore, in this way, it can improve the completeness of detection and consistency.

## 2. Materials and Methods

### 2.1. Dataset Acquisition and Processing

The road crack dataset used in this study was collected by our team in 2024 using low-altitude UAV imaging. We used a multirotor UAV equipped with a high-resolution RGB camera so that crack details could be clearly captured. The dataset contains 800 original images in total. These images cover different pavement types, such as asphalt and concrete. They also include cracks at different scales and under various complex background conditions. To increase data diversity, we collected the images under different lighting conditions and in different environments. We then divided the dataset into 600 training images, 100 validation images, and 100 test images. During annotation, we used LabelMe to manually label the images. After loading the original JPG images, the annotators drew rectangular bounding boxes around the crack regions. They saved the annotations in JSON format. We then converted the annotation files into the format required for object detection, such as the YOLO format. We also matched each annotation file with its corresponding image for later model training. During preprocessing, we resized all images to 640 × 640 and normalized them to fit the network input size and improve training efficiency. The preprocessed images and their corresponding label files were then used as the input data for model training and evaluation. In addition to visual annotations, each image is associated with a corresponding textual description to provide crack-related semantic information. It should be noted that these textual descriptions are automatically generated based on crack annotations rather than manually annotated. Specifically, the text generation process first analyzes crack bounding box annotations to determine crack existence and spatial distribution characteristics within each image. Then, crack-related attributes are automatically extracted, including morphological characteristics (e.g., slenderness, continuity, fragmentation patterns, and orientation variations) as well as structural properties (e.g., local complexity and overall distribution patterns). Finally, predefined semantic templates are used to convert these extracted attributes into natural-language descriptions, thereby generating semantic representations corresponding to each image. These automatically generated textual descriptions provide high-level semantic prior information and serve as semantic guidance signals in the multimodal framework, helping improve discrimination between crack and non-crack regions under complex background conditions.

### 2.2. MCGC-Net

**Figure 2:**
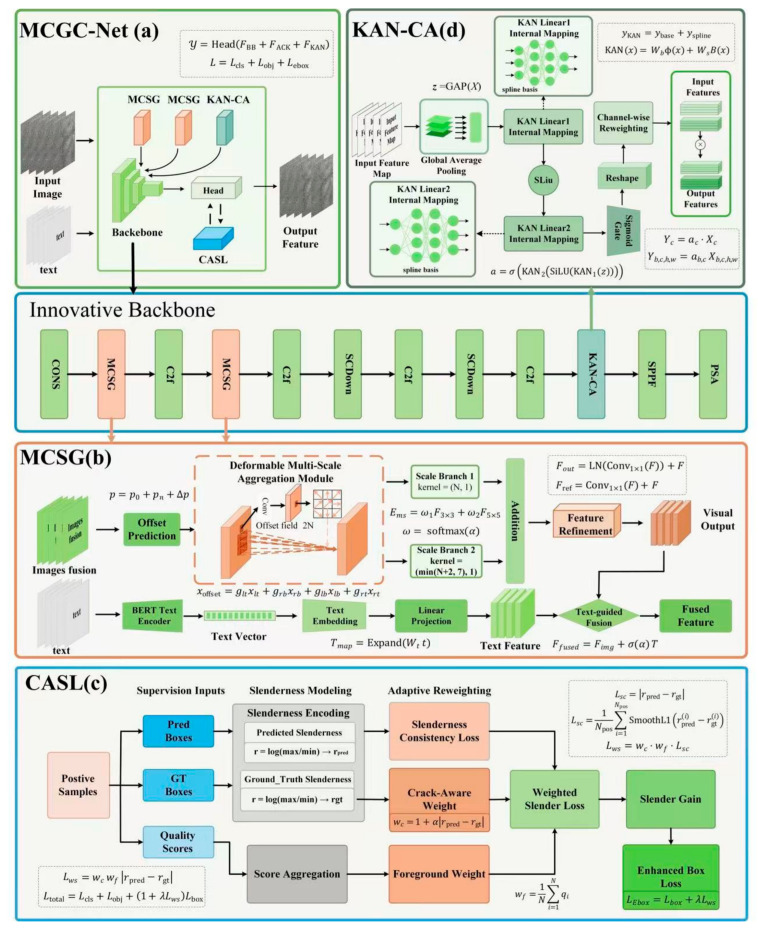
(**a**) Overall structure of MCGC-Net; (**b**) Structure of the MCSG module; (**c**) Structure of the CASL module; (**d**) Structure of the KAN-CA module.

The proposed MCGC-Net is built on the YOLOv10 framework. First, the network takes both UAV road images and crack description texts as input. In this way, the model builds a multimodal detection framework that combines visual information with semantic information, as shown in Figure 2a. This design helps the model better represent both the appearance features and the high-level semantic information of road cracks. Then, the model adds a Multimodal Contrastive Semantic Gating (MCSG) module in the high-level feature learning stage, as shown in Figure 2b. This module combines text semantics with the semantic differences between images and texts within the same batch. This design helps the module guide visual features in a more direct way. With this design, the module can adjust channel responses more flexibly. This helps the model separate crack regions from complex backgrounds more effectively. Figure 2c shows that the model also introduces a Crack-Aware Slenderness Loss (CASL) in the bounding box regression stage. CASL directly keeps the aspect ratio of the predicted boxes consistent with that of the ground-truth boxes. This design helps the model keep the geometric shape of slender cracks more accurately. As a result, the model can locate small cracks with blurred boundaries more accurately. At the feature enhancement stage, the model also adds a KAN-based Nonlinear Channel Attention (KAN-CA) mechanism, as shown in Figure 2d. To better match the fracture structure that shows thin extension under nonlinear mappings, higher orders can be added. Due to this design, the model will be able to generate higher quality and consistent detections. MCGC-Net introduces only limited additional computational overhead while maintaining a practical detection efficiency architecture. In addition to increasing the detection accuracy, the precision of locating and structure representation shows improvement for road cracks in complicated background environments. This improvement makes the model better suited for precise road crack detection in UAV images.

#### 2.2.1. Multimodal Contrastive Semantic Gating Module (MCSG)

In UAV-based road crack detection, complex background environments affect model recognition. Shadow, oil stain, wheel ruts, water mark and other phenomena have similarities with real crack textures under gray conditions, including edge shapes and localized texture responses. Due to this reason, the detector will mistakenly identify background interference as cracks in a high-level feature space. There will be problems of false alarms or mislocated bounding boxes. Previous studies have enhanced the representations of visual features by means such as convolutions, multi-scale fusion and anisotropic attention. Nevertheless, these methods are primarily based on texture and structure information of the visual domain. They still lack explicit constraints from high-level semantic priors. As a result, there is still difficulty in mitigating the degree of misidentification between crack and non-crack areas under complicated backgrounds.

Therefore, an MCSG multi-modal semantic contrastive gating mechanism is proposed to resolve this problem. MCSG does not enhance the feature of cracks by appending an attention augmentation branch at the end of the visual representation; rather, it leverages global information. Instead, it follows a unified pipeline of semantic guidance, cross-modal fusion, and contrastive modulation, where adaptive spatial modeling, multi-scale feature extraction, textual semantic guidance, and in-batch semantic difference modeling are integrated into a continuous feature enhancement process. Without introducing any additional contrastive loss or modifying the main structure of the original detector, MCSG performs adaptive channel recalibration under cross-modal semantic constraints, thereby improving the model’s ability to distinguish real crack targets from confusing background patterns.

Let the visual feature input to the MCSG module be $X\in R^{B\times C_{in}\times H\times W}$, where B is the batch size, $C_{in}$ is the number of input channels, and H and W denote the spatial dimensions of the feature map. Since crack structures are usually slender, irregular in direction, and often submerged in complex background textures, directly applying convolution on a fixed sampling grid can easily be affected by background noise and locally redundant textures. To alleviate this problem, adaptive spatial modeling is first introduced. Specifically, a convolution layer is used to predict the spatial offset field, as shown in Equation (1). The predicted offsets are then added to the regular sampling grid $P_{0}$ to generate adaptive sampling coordinates, as shown in Equation (2). Finally, use a bilinear interpolator to upsample the input feature map as per Equation (3).

(1) $$\Delta P={Conv}_{3\times 3}(X)$$

(2) $$P=P_{0}+\Delta P$$

(3) $$X_{off}=Bilinear(X,P)$$

Adaptive shifting of sampling positions based on the expected response to cracks in this study is fundamentally different from the simple expansion of the receptive field. Thus, during this time, there were no longer any limitations to the selection of the grids, so as not to focus too much on long cracks or eliminating other factors such as complicated texture and noise. That is to say, at this stage, it provides a clearer reference for subsequent semantics improvement, but does not impose severe semantic restrictions on the combined and noisily polluted responses. Based on these resampled features, we then add multiple-scale convolution branches to enhance their crack representation scale by scale. Two convolution branches of different sizes are introduced to obtain the feature map expressions given by Equation (4). The two branches are then dynamically connected with a certain weight parameter using Equation (5).

(4) $$F_{1}={Conv}_{1}(X_{off}),F_{2}={Conv}_{2}(X_{off})$$

(5) $$F=\omega_{1}F_{1}+\omega_{2}F_{2},[\omega_{1},\omega_{2}]=Softmax(\theta )$$

This process cannot integrate features from different scale levels simultaneously, but rather tries for a static equilibrium of fine-grained crack textures with large-scale contextual structure. Because of the very small width of cracks, they can be preserved better when selecting a smaller receptive field in such situations. For crack regions with large areas of damage and irregular directions, a larger-scale branch may be necessary to supplement the context. Thus, adaptive fusion using learnable weights can automatically optimize the emphasis in multi-scale data to meet the requirements for identifying crack objects with varying sizes or complex forms across different situations. After normalization, non-linear activation and lightening improvements are applied to obtain a visually enhanced feature given by Equation (6).

(6) O=Φ(F)

However, relying only on the visual branch is still insufficient to fundamentally solve semantic confusion in complex backgrounds. To this end, we further introduce automatically generated crack semantic descriptions derived from crack annotations using predefined semantic templates as a source of high-level semantic priors. These descriptions summarize crack morphology, spatial distribution patterns, and structural characteristics that cannot be explicitly captured by visual appearance alone. Let $S_{i}$ denote the text description of the i-th sample. A pretrained language model is first used to encode the description into a textual semantic vector, as shown in Equation (7). The text feature is then projected into the visual channel space, expanded to the spatial dimension, and fused with the visual feature, as shown in Equation (8).

(7) $$t_{i}=BERT(S_{i})\in R^{C}$$

(8) $$F_{m,i}=O_{i}+\lambda T_{i}$$

Here, λ is a learnable fusion coefficient, and $T_{i}$ denotes the spatially mapped form of the text feature. The purpose of this design is not to replace visual features with textual semantics, but to provide a more stable high-level semantic reference for visual representations. This helps the model establish clearer discrimination when encountering regions that look locally similar in texture but differ in semantic meaning. Put differently, the text branch provides prior knowledge about what a crack is, while the visual branch preserves local appearance cues about what a crack looks like. Their combination can therefore reduce the ambiguity caused by relying solely on visual appearance. After cross-modal fusion, simply injecting textual semantics as a static enhancement signal is still not enough to capture semantic differences between the current sample and other samples in the same batch. For this reason, we further introduce an in-batch semantic contrastive modulation mechanism. First, global average pooling is applied to the fused feature to obtain an image-level representation, as shown in Equation (9). The image and text representations are then normalized, as shown in Equation (10). Based on these normalized features, an in-batch image–text similarity matrix is constructed, as shown in Equation (11). After excluding the matched text of the current sample, the remaining text features in the batch are aggregated with softmax-normalized weights to form a negative semantic prototype ${\hat{t}}_{i}^{-}$, from which a semantic difference vector is constructed, as shown in Equation (12).

(9) $$g_{i}=GAP(F_{m,i})$$

(10) $${\hat{g}}_{i}=\frac{g_{i}}{∥g_{i}{∥}_{2}},{\hat{t}}_{i}=\frac{t_{i}}{∥t_{i}{∥}_{2}}$$

(11) $$s_{ij}=\frac{{\hat{g}}_{i}^{⊤}{\hat{t}}_{j}}{\tau }$$

(12) $$v_{i}={\hat{t}}_{i}-{\hat{t}}_{i}^{-}$$

The significance of this design is that the model no longer focuses only on the semantics of the current text description itself, but also on how that semantics differs from others within the batch. In this sense, the semantic difference vector characterizes the uniqueness of the current sample in the in-batch semantic distribution, allowing the subsequent channel modulation process to emphasize semantic dimensions that are more discriminative for crack recognition, rather than treating all textual information as equally important static guidance. Based on this semantic difference, an element-wise interaction is used to generate the channel gating input, as shown in Equation (13). A lightweight mapping network is then used to produce channel-wise gating coefficients, as shown in Equation (14). Finally, the fused feature is adaptively recalibrated at the channel level to obtain the enhanced output feature, as shown in Equation (15).

(13) $$u_{i}={\hat{g}}_{i}⊙v_{i}$$

(14) $$\gamma_{i}=\sigma (MLP(u_{i}))$$

(15) $$Y_{i}=F_{m,i}⊙\gamma_{i}$$

Unlike conventional attention mechanisms, which generate weights directly from visual statistics, the gating coefficients in our method use more than just the current visual response. In addition, there is a sense of disparity between the current sample and the negative-semantic prototype within the same batch. Therefore, in this way, the gating process can be considered to be adjusting channels through contrastive semantical guidance. It strengthens the feature channels that are more consistent with crack semantics. At this time, it also reduces some weak channel-background interference that has a localized feature and is semantically close to the negative sample. Therefore, it is more likely to eliminate false alarms in such complicated background environments.

Adaptive spatial sampling, multi-scale visual enhancement, textual semantic fusion and in-batch semantically contrasting gating are the main components of the MCSG network. This part’s significance is not in the addition of text description or the installation of an additional attention block but instead in providing such high-level semantic reference and in-batch semantic difference constraint information within the feature enhancement procedure. Therefore, it is possible to identify real cracks among visually similar background areas in complicated UAV remote sensing images more accurately through this method. This design can both strengthen the meaning representation of crack areas and reduce false recognitions due to shadows, oil residues, wheel tracks or other complicated surface characteristics. Because of this, the module provides more stable and more discriminative features for the following detection head. In addition, the module works entirely at the feature level and does not rely on extra contrastive loss functions or structural reconstruction. Therefore, it maintains good compatibility with existing detection frameworks and is also highly extensible.

#### 2.2.2. Crack-Aware Slenderness Loss (CASL)

In UAV-based road crack detection, crack targets usually have clear structural features. These targets are often very narrow and slender, and their boundaries are often blurred. In many cases, cracks in one image are only a few pixels wide. Because of this, conventional overlap-based regression losses, such as IoU and CIoU, are very sensitive to small width errors. Even a slight change in the predicted width can greatly reduce the overlap between the predicted box and the ground-truth box. This change often causes a large variation in the regression loss. As a result, the optimization process can become unstable, and the predicted boxes may turn out too wide or too short. This problem can further cause inaccurate localization and missed detection of small cracks.

To solve this problem, we propose a Crack-Aware Slenderness Loss (CASL). This loss is different from methods that only adjust the regression weight based on the original IoU-based loss. CASL does not simply enlarge the bounding box error. Instead, CASL is designed around three main ideas. The first idea is to enhance slenderness consistency for crack-oriented bounding box regression. The second idea is to strengthen the learning of slender samples in an adaptive way. The third idea is to keep the optimization process stable while considering prediction quality. CASL starts from the aspect ratio consistency of crack targets. It introduces an additional crack-aware slenderness consistency constraint into the regression process to the bounding box regression process. With this design, the model can better maintain slenderness consistency during crack localization while keeping its original localization ability.

We define the predicted bounding boxes and their corresponding ground-truth boxes in the positive sample set as shown in Equation (16). We represent each bounding box as shown in Equation (17). We then calculate the width and height of each box as shown in Equation (18). To provide an approximate geometric cue for slender crack targets, we introduce a logarithmic slenderness measure based on the bounding box aspect ratio, as shown in Equation (19).

(16) $$B_{p}=\{b_{p}^{\left(i\right)}{\}}_{i=1}^{N},B_{g}=\{b_{g}^{\left(i\right)}{\}}_{i=1}^{N}$$

(17) $$b=(x_{1},y_{1},x_{2},y_{2})$$

(18) $$w=x_{2}-x_{1},h=y_{2}-y_{1}$$

(19) $$r(b)=log\left(\frac{max(w,h)+\varepsilon }{min(w,h)+\varepsilon }\right)$$

Here, ε is a numerical stability term. In addition, the purpose of this Design is to calculate the aspect ratio. After taking a log of the data, significant ratios become more linearly correlated with subsequent outcomes for thinner objects such as cracks. Direct modeling of the original aspect ratio would be overly sensitive to small deviations in pixels; after applying this transformation, changes in slenderness are perceived less sensitively by the loss function. Additionally, it will provide a basis for carrying out subsequent calculations. We can use the obtained slenderness value to calculate a shape consistency loss function of predicted boxes and their corresponding ground-truth box positions, given by Equation (20).

(20) $$L_{s}^{\left(i\right)}=SmoothL1(r(b_{p}^{\left(i\right)}),\;r(b_{g}^{\left(i\right)}))$$

It is no longer the same as bounding box regression loss. An additional restriction exists above the requirement for position overlap. It encourages the predicted box to maintain a similar slenderness level to the ground truth box; i.e., although it has moved relatively near to the goal post, CASL will penalize predictions that significantly deviate in slenderness consistency, such as overly wide or overly narrow boxes. We attempt to help the model learn not only the right box position but also details such as the thinness of a crack. Therefore, the proposed loss helps improve the consistency of bounding box shapes for relatively slender crack targets; it does not focus primarily on overlapping conditions. Different crack samples can be of extremely diverse slenderness. If the same shape restriction is imposed on all samples, it will be difficult for optimization to improve true slender-crack performance. To address this problem, we further introduce a crack-aware weighting factor:

(21) $$k_{i}=Clamp\left(\frac{r(b_{g}^{\left(i\right)})}{\tau },\;0,\;k_{max}\right)$$

Here, τ is the slenderness adjustment coefficient, and $k_{max}$ is the upper limit of the weight. Due to a more slender ground-truth box, its coefficient is higher. This design aims to alter the strength of geometric constraints according to how slender the target is; thus, the optimization process can pay more attention to cracks whose widths deviate significantly and structural distortions occur. Compared with a fixed-weight strategy, this adaptive weight based on slenderness is more suitable for crack detection. It also gives more regression focus to samples that need stronger geometric preserver box-optimization processes. It is also influenced by how well the matches are matched if the model treats all positive samples in the same way. Low-quality matches or samples with unclear boundaries may add unnecessary interference to the loss direction. For this reason, we further introduce a quality-aware weight:

(22) $$\omega_{i}=\sum_{c=1}^{C}s_{\left(i,c\right)}$$

Here, $s_{\left(i,c\right)}$ denotes the soft label score produced by the assigner. Therefore, this type can modify CASL without regard for either the shape parameters or the accuracy of sample matching data when adjusting the length parameter. In other words, the target has been selected as a combination of clear thinness and high-confidence levels that can enhance the weight in training. At this time, lower quality data will have less effect on it. To reduce interference caused by noisy samples, indistinct boundaries of objects, etc., hard match problems help improve the overall stability of the model construction in this process. By combining the two weighting terms above, CASL can be written as in Equation (23):

(23) $$L_{CASL}=\frac{1}{S}\sum_{i=1}^{N}\omega_{i}\cdot k_{i}\cdot L_{s}^{\left(i\right)}$$

Here, $S=\sum_{i}\omega_{i}$ is the normalization factor. In this form, CASL brings three types of information into one loss term. These include shape consistency constraint, slenderness-aware weighting, and sample-quality modulation. Because of this, the bounding box optimization process becomes better adapted to slender crack localization characteristics, instead of treating all samples as the same kind of object. Finally, CASL is added to the original bounding box regression loss as a regularization term, which gives the revised regression objective shown in Equation (24):

(24) $$L_{box}^{*}=L_{IoU}+\lambda L_{CASL}$$

Although CIoU also introduces an aspect-ratio consistency term, its primary objective is general bounding box regression optimization. In contrast, the proposed CASL is specifically designed for crack detection scenarios and focuses on adaptive slenderness consistency for elongated crack targets. Unlike the fixed aspect-ratio penalty used in CIoU, CASL further incorporates slenderness-aware weighting and quality-aware modulation to improve localization stability under challenging crack conditions. Here, *λ* is the balancing coefficient. This design does not completely replace the original IoU-based loss with CASL. Instead, this design adds an extra geometric constraint and still keeps the original overlap-based optimization ability. In this way, the model learns not only to place the predicted box in the right position, but also to maintain localization consistency for slender crack targets. So, CASL and conventional IoU-based losses are not two separate choices. They work together in the regression process. CASL helps the model preserve the slender characteristics of crack targets, while the IoU-based loss focuses on how well the predicted box matches the target position. Together, they give crack detection box regression a more complete constraint.

Overall, CASL is not simply adding an extra term for aspect ratio. Instead, CASL introduces a crack-oriented slenderness consistency regularization strategy for crack localization and focuses on improving the regression stability of slender structures. CASL provides a coarse geometric prior for crack structures. It should be noted that the proposed slenderness measure is a simplified approximation and cannot fully describe complex crack geometries, such as curved, diagonal, or irregular crack structures. It also combines adaptive enhancement for slender samples with stable optimization that considers prediction quality. This design helps CASL reduce localization instability caused by slenderness variation and improve localization stability. Thus, the problem of missing detection and box distortion in small-target scenario scenes will be reduced to some extent. At the same time, it will work only at the level of losses. It does not add any new network branches or modify the original model structure. Therefore, such methods are easy to incorporate into the existing detection frameworks without significant performance loss.

#### 2.2.3. KAN-Based Nonlinear Channel Attention (KAN-CA)

In UAV-based road crack detection, road crack appearance may vary significantly under different imaging conditions and pavement environments. Their directions vary greatly, the shapes of their branches are generally irregular, and the width and orientation at different places might be different. As a result, crack-related feature responses may exhibit complex variations under different crack conditions and background interference. At the same time, crack-related features can show very different response strengths in different regions. This problem is more pronounced in regions with turns or local breaks and sharp changes in form; then, the feature representation will be unstable. This instability may also reduce the reliability of the final detection results’ integrity and uniformity. Existing channel attention-based approaches have enhanced the significance of some responses relatively. However, most of these methods still rely on linear transformations or relatively simple mapping functions in the channel projection process. Because of this, their flexibility in modeling complex channel interactions under challenging crack scenarios is still limited. Therefore, in order to improve the flexibility of channel interaction modeling under challenging crack conditions without introducing a significant computational burden, it is beneficial to incorporate a more flexible nonlinear mapping mechanism at the channel level so that channel responses can be recalibrated in a more adaptive manner.

To address this problem, we propose a KAN-based Nonlinear Channel Attention (KAN-CA) mechanism. Unlike conventional channel attention methods, which usually generate channel weights through a simple linear squeeze-and-recover process, KAN-CA does not simply reweight channel statistics. Instead, KAN-CA combines channel statistical description, nonlinear high-order relation modeling, and adaptive channel recalibration in one process. KAN-CA does not modify the original detection framework or introduce any extra loss function. By using spline-based mappings, KAN-CA improves the representation of complex channel dependencies. In this way, KAN-CA enhances the stability of feature representation under challenging crack conditions, including slender, discontinuous, and irregular crack patterns.

Let the input feature to this module be $X\in R^{B\times C\times H\times W}$, where B is the batch size, C is the number of channels, and H and W denote the spatial dimensions of the feature map. Since the structural information of crack targets is unevenly distributed across the spatial dimension, directly applying a complicated nonlinear mapping to the original feature map would not only incur considerable computational cost, but also make it difficult to highlight the overall response characteristics at the channel level. Therefore, global average pooling is first applied to the input feature to extract a global statistical description for each channel, as given in Equation (25):

(25) $$z=GAP(X)\in R^{B\times C}$$

The purpose of this step is not only to compress the spatial dimension. This step also aggregates channel-response information from different spatial positions into the channel domain, so that each channel can form a more stable global descriptor. In this way, the model can preserve the overall trend of structural responses and reduce the influence of local noise and spatial disturbance on the later channel modeling process. This step provides a more compact and more stable input for nonlinear relation modeling. In conventional channel attention structures, channel compression and channel recovery are usually implemented with two linear transformations. Although this design is computationally efficient, it is often not sufficient for modeling complex nonlinear channel relationships under challenging crack-response conditions. To improve nonlinear modeling ability, we replace the standard linear mapping with a KAN structure. Specifically, we first project the channel statistical vector into a low-dimensional latent space, as shown in Equation (26):

(26) $$h=\phi ({KAN}_{1}(z))$$

Here, ϕ(⋅) denotes a nonlinear activation function. This step is not used as a simple feature compression operation. Instead, this step uses the nonlinear fitting ability of KAN to reorganize the response relationships among different channels in the latent space. Compared with conventional linear mappings, this strategy can model more flexible channel-response relationships under varying crack conditions in a more flexible way. Because of this, the channel representation becomes more suitable for the later weight generation process. In addition, the KAN mapping includes both a base linear term and a spline expansion term, and it can be written as in Equation (27):

(27) $$KAN(x)=W_{b}\;\sigma (x)+W_{s}\;B(x)$$

where $W_{b}$ denotes the base weight, B(x) is the basis expansion term constructed using *B*-splines, and $W_{s}$ represents the spline weight parameter. Unlike traditional linear mappings, which can only capture linear correlations between channels, this structure is derived from the Kolmogorov–Arnold representation principle and can approximate complex nonlinear relationships with high precision through the composition of one-dimensional functions. In other words, the advantage of KAN is not simply that it is “more complex,” but that it can more finely describe the potential high-order coupling relationships between channels. This property enables the model to capture more flexible nonlinear channel dependencies and improve feature adaptation under challenging feature-response variations caused by complex crack appearances. After obtaining the latent representation, it is further mapped back to the original channel dimension to produce a channel modulation signal corresponding to the input feature, as shown in Equation (28). A sigmoid activation is then applied to obtain normalized channel-wise coefficients, as shown in Equation (29):

(28) $$w={KAN}_{2}(h)\;$$

(29) α=σ(w)

The purpose of this process is to convert the enhanced nonlinear channel relationships learned in the latent space into channel weights that can act directly on the original feature. Unlike standard attention mechanisms, which generate weights directly from linear mapping outputs, the coefficients here are obtained after nonlinear high-order relation modeling. Because of this, these coefficients can better reflect the relative importance of different channels under varying crack-response patterns. This also makes the following channel recalibration more targeted and more effective. Finally, we reweight the input feature channel by channel using the generated coefficients, and this gives the enhanced output feature shown in Equation (30):

(30) Y=X⊙α 

Here, the equation denotes channel-wise multiplication. Through this process, the model is able to dynamically adjust its sensitivity levels for all components based on varying feature-response conditions. The part giving more attention to those channels that are more sensitive to discriminative crack-related feature responses at the same time reduces its influence over other types of data, such as background noise interference, repetition texture disturbance, and unstable changes in structural features. To some extent, this process can help the model maintain more stable feature representation under challenging crack conditions and maintain a relatively stable representation form for features during crack bends or local interruptions.

In summary, the KAN-CA module is composed of four parts: extracting channel statistical features; performing KAN-based non-linear latent-space mapping; generating channel weights; and adaptive selection of channels. The core issue of this part is not only to replace KAN in the channel attention block as an instance of mapping. Therefore, the module adds more nonlinear representation capability to the channel model directly. Thus, the network can learn more flexible nonlinear channel dependencies and improve feature representation adaptability under challenging crack scenarios. This design helps improve the robustness of feature representation for slender, discontinuous, and irregular crack patterns. At the same time, this module does not alter the initialization of the detection model and introduces no additional loss terms or structural-reconstruction modules. As for the mapping work primarily done in the compressed channel space, it has relatively low computational cost and maintains the high recognizability and scalability of this module.

## 3. Experiments and Discussion

### 3.1. Experimental Setting

All the experimentations in this paper were performed under a unified hardware-software configuration to ensure that the results are more credible and reduce interference from external factors. The AutoDL platform offered all required hardware support. We have used the same computer to carry out all of our experiments; thus, any variations brought about by this reason were negligible. There are no other problems here—using the exact same programming environment for each set of tests, it is reasonable that both OS platforms would run stably at identical software versions too. Hence, these two factors might bias the results significantly. Table 1 shows the specific hardware and software configurations.

### 3.2. Evaluation Metrics Overview

To quantify the performance of the system using these four parameters, precision, recall, F1-score, and map@50, these metrics were used to assess the performance of the UAV-based road crack detection model in a more complete way. As shown in Equations (31)–(34), these metrics reflect the model’s detection ability and localization accuracy from different aspects. Specifically, true positive (TP) refers to the number of crack targets that the model detects correctly. False positive (FP) refers to the number of regions that are predicted as cracks but are actually non-crack areas. False negative (FN) refers to the number of crack targets that exist in the image but are not detected by the model.

(1) Precision: Precision measures how many regions predicted as cracks are actually crack targets. This metric reflects the accuracy of the detection results. It is defined as follows:

(31) $$Precision=\frac{TP}{TP+FP}\;$$

(3) Recall: Recall measures the proportion of actual crack targets that are correctly detected by the model, reflecting the model’s ability to cover the target objects. It is defined as follows:

(32) $$Recall=\frac{TP}{TP+FN}$$

(5) F1-score: The F1-score is the harmonic mean of Precision and Recall. This metric shows the balance between detection accuracy and detection completeness. It is defined as follows:

(33) $$F1=\frac{2\times Precision\times Recall}{Precision+Recall}=\frac{2TP}{2TP+FP+FN}$$

(7) AP_50_: Average Precision (*AP*) represents the average precision for a single class. Mean Average Precision (mAP) is the average of *AP* values across all classes. This metric reflects the overall performance of an object detection model in multi-class tasks. The formulas are given as follows:

(34) $$AP=\int_{0}^{1}P(r)dr$$

### 3.3. Module Effectiveness Experiments

#### 3.3.1. Effectiveness of the MCSG

We replaced the standard 3 × 3 convolution in the baseline network with deformable convolution (DCNv2) [19], dynamic convolution (DyConv) [20], MCSG without contrastive gating, MCSG without multi-scale aggregation, and the complete MCSG, respectively, to evaluate the effects of multi-scale aggregation and contrastive gating on crack detection performance.

As shown in Table 2, the baseline model achieved 81.25% Precision, 74.73% Recall, 77.85% F1-score, and 80.65% AP_50_. After the model replaced the standard convolution with DCNv2, Precision, Recall, and F1-score increased to 82.68%, 75.94%, and 79.17%, respectively. However, AP_50_ dropped to 78.92%. The model used DyConv, Precision, Recall, and F1-score reached 82.45%, 75.62%, and 78.89%, respectively. However, AP_50_ further dropped to 78.64%. These results show that deformable sampling and dynamic convolution can improve local feature modeling to some extent. However, these methods still cannot effectively improve the overall localization quality of crack targets.

By comparison, the MCSG variant without contrastive gating achieved 83.67% Precision, 77.79% Recall, 80.62% F1-score, and 82.70% AP_50_. These values were 2.42%, 3.06%, 2.77%, and 2.05% higher than the baseline, respectively. This result shows that the multi-scale aggregation strategy can effectively improve the representation of crack features at different scales. The MCSG variant without multi-scale aggregation achieved 83.12% Precision, 76.58% Recall, 79.72% F1-score, and 82.14% AP_50_. These values were 1.87%, 1.85%, 1.87%, and 1.49% higher than the baseline, respectively. This result shows that the contrastive gating mechanism can also bring stable performance gains.

The complete MCSG achieved the best overall performance. Its Precision, Recall, F1-score, and AP_50_ reached 84.31%, 76.97%, 80.47%, and 83.72%, respectively. Compared with the baseline, the improvements were 3.06%, 2.24%, 2.62%, and 3.07%. Compared with the version without contrastive gating, Precision and AP_50_ further increased by 0.64% and 1.02%, respectively. Compared with the version without multi-scale aggregation, the improvements were 1.19%, 0.39%, 0.75%, and 1.58%, respectively. These results show that multi-scale aggregation and contrastive gating work well together. Their combination is more effective for improving both the accuracy and the localization performance of crack detection.

#### 3.3.2. Effectiveness of the CASL

Based on the baseline v10DetectionLoss, we replaced the bounding box regression loss with CIoU Loss [21], EIoU Loss [22], Focal-EIoU Loss [23], and different settings of CSLS Loss to study how geometric prior constraints affect crack detection performance. In all experiments, we kept the classification loss weights and training settings the same so that the comparison would be fair.

As shown in Table 3, the baseline CIoU Loss achieved 81.25% Precision, 74.73% Recall, 77.85% F1-score, and 78.63% AP_50_. After replacing it with the EIoU loss function, the above accuracy reached 81.68–75.12%, 78.26%, and 79.14%. The separation of modeling for the width and height gives a more accurate result of cracks. Focal-EIoU loss improved Precision and Recall to 82.41% and 75.92%, respectively, but its mAP@50 decreased to 78.51 per cent. It can be concluded that increasing attention to the “hard” ones has failed to enhance generalization effects.

After introducing the slenderness restriction, the CSLS loss reached 83.12% Precision, 76.45% Recall, 79.65% F1-score and 78.62% mAP@50. Different kernel-size settings have shown distinct results in performance. In particular, CSLS loss (3 × 3) achieved 83.61% Precision, 76.24% Recall, 79.76% F1-score, and 81.42% AP_50_. Compared with the baseline CIoU loss, the improvements were 2.36%, 1.51%, 1.91%, and 2.79%, respectively. This result shows that local slender-geometry constraints are more effective in improving the regression quality of crack targets. By comparison, CSLS loss (5 × 5) achieved 80.34% AP_50_. Although this result was still better than the baseline, it was lower than the 3 × 3 setting. This suggests that a larger kernel scale may reduce the sensitivity to the local geometric characteristics of small cracks.

The full model achieved the best overall performance. Its Precision, Recall, F1-score, and AP_50_ reached 84.15%, 76.58%, 80.19%, and 82.13%, respectively. Compared with the baseline CIoU loss, these values increased by 2.90%, 1.85%, 2.34%, and 3.50%. Compared with the best single loss setting, namely CSLS loss (3 × 3), these values further increased by 0.54%, 0.34%, 0.43%, and 0.71%, respectively. These results show that the geometric prior constraint on crack slenderness can effectively improve bounding box regression quality. When the model uses this design together with the full detection framework, it can further improve detection accuracy and localization stability for slender crack targets.

#### 3.3.3. Effectiveness of the KAN-CA

In the baseline YOLOv10n, we inserted SE-Net [24], ECA [25], DSConv [26] and different parameter settings of the proposed KAN-CA module into the BottleNeck layer, while keeping all other training settings unchanged, in order to evaluate the effect of nonlinear channel modeling on crack detection performance. DSConv, originally proposed for tubular structure segmentation through topological geometric constraints, was included as a representative geometry-aware convolution operator for comparison [27].

As shown in Table 4, the baseline model achieved 81.25% Precision, 74.73% Recall, 77.85% F1-score, and 80.65% AP_50_. After SE-Net was added, these metrics increased to 82.15%, 75.38%, 78.62%, and 81.42%, respectively. When ECA was used, these values further increased to 82.47%, 75.61%, 78.89%, and 81.68%, respectively. These results show that conventional channel attention mechanisms can improve crack-related feature responses to a certain degree. This improvement further leads to better detection performance.

By comparison, the KAN-based nonlinear channel modeling gave better results. KAN-CA (grid = 3, order = 2) reached 83.28% Precision, 76.45% Recall, 79.72% F1-score, and 82.67% AP_50_. These values were 2.03%, 1.72%, 1.87%, and 2.02% higher than the baseline, respectively. KAN-CA (grid = 7, order = 4) reached 82.95% Precision, 76.89% Recall, 79.81% F1-score, and 82.45% AP_50_. These values were 1.70%, 2.16%, 1.96%, and 1.80% higher than the baseline, respectively. These results show that KAN-CA improves the model’s ability to represent complex crack structures through spline-based nonlinear channel mapping.

In addition to accuracy improvement, we further analyze the computational complexity shown in Table 4. Compared with the baseline model (21.7 GFLOPs and 8.9M parameters), SE-Net and ECA introduce relatively small increases in computational cost, while KAN-CA results in higher FLOPs and parameter counts due to the additional spline-based nonlinear mapping operations. Specifically, KAN-CA (grid = 3, order = 2) increases the computational complexity to 47.2 GFLOPs and 20.5M parameters, and similar trends are observed for higher-order configurations. Although the proposed KAN-CA introduces additional computational overhead, the nonlinear mapping is mainly performed on compressed channel descriptors after global average pooling rather than on full-resolution spatial features. Therefore, the additional complexity is mainly concentrated in channel-level feature interaction. Overall, the experimental results indicate that KAN-CA can improve feature representation ability under challenging crack conditions while introducing a moderate increase in computational complexity.

The complete method achieved the best overall performance. Precision, Recall, F1-score, and AP_50_ reached 83.36%, 77.45%, 80.30%, and 83.34%, respectively. Compared with the baseline, these values improved by 2.11%, 2.72%, 2.45%, and 2.69%, respectively. Compared with SE-Net, the gains were 1.21%, 2.07%, 1.68%, and 1.92%, respectively, while compared with ECA, the improvements were 0.89%, 1.84%, 1.41%, and 1.66%. In addition, compared with the two individual KAN-CA configurations, the full method still achieved the best Precision, F1-score, and AP_50_, indicating that the proposed KAN-CA module works well together with the overall detection framework and can further improve the representation of slender, discontinuous, and irregular crack structures.

### 3.4. Ablation Experiments

As shown in Table 5, when no module was introduced, the baseline model achieved 81.25% Precision, 74.73% Recall, 77.85% F1-score, and 80.65% AP_50_. After introducing multimodal input alone, these metrics increased to 82.55%, 75.41%, 78.37%, and 81.69%, respectively, indicating that textual semantic information can enhance the discriminative ability of the model for crack targets to a certain extent and provide a more favorable feature basis for the subsequent modules.

In the single-module experiments, all three modules brought performance gains to different degrees. Among them, MCSG produced the most significant improvement. Based on multimodal input, it further increased Precision, Recall, F1-score, and AP_50_ to 84.91%, 77.24%, 80.89%, and 84.21%, respectively, representing gains of 2.36%, 1.83%, 2.52%, and 2.52% over the multimodal-input-only setting. As shown in the above, using MCSG helps improve the recognition of cracks over other regions in image data. When CASL was used alone, Precision, Recall, F1-score, and AP_50_ reached 84.31%, 76.82%, 80.39%, and 82.13%, respectively. This result shows that geometric constraints can steadily improve the localization quality of crack targets. When KAN-CA was used alone, Recall reached 77.79%, and F1-score and AP_50_ reached 80.89% and 83.70%, respectively. This result also shows that nonlinear channel modeling can improve crack-region coverage and overall detection performance. The results of the dual-module combinations further show that the proposed modules work well together. The combination of Multimodal + MCSG + CASL achieved the best Precision and F1-score, reaching 88.81% and 83.30%, respectively. Its Recall and AP_50_ also reached 78.43% and 85.18%. This result shows that semantic guidance and geometric constraints can improve feature discrimination and also make bounding box regression more stable. The combination of Multimodal + CASL + KAN-CA also achieved strong results. Its Recall reached 78.68%, and its AP_50_ reached 85.71%. These two values were the highest Recall and AP_50_ among the three dual-module settings. This result suggests that this combination performs better in crack coverage and overall detection performance. By comparison, the Multimodal + MCSG + KAN-CA setting achieved an AP_50_ of 85.00% and an F1-score of 81.46%. This setting still performed better than the single-module settings. However, its overall improvement was smaller than that of the other two dual-module combinations.

When the model used all three modules together, it showed the best overall performance. The model achieved 89.55% Precision, 79.40% Recall, 84.17% F1-score, and 86.58% AP_50_. Compared with the baseline, these values increased by 8.30%, 4.67%, 6.32%, and 5.93%, respectively. Compared with the multimodal-input-only setting, these values increased by 7.00%, 3.99%, 5.80%, and 4.89%, respectively. These results show that the performance improvement did not come from only one module. It came from the joint effect of multimodal input, MCSG, CASL, and KAN-CA in semantic discrimination, geometric constraint, and structural representation. Among the single-module settings, MCSG brought the most direct improvement. CASL mainly improved the localization stability of crack targets. KAN-CA further strengthened the representation of cracks with complex structures. Working simultaneously at this time to produce an appropriate model results in better stabilities for detection accuracy, object recognition rates, as well as generalized performance levels.

### 3.5. Comparison Experiments with Other Networks

**Figure 3:**
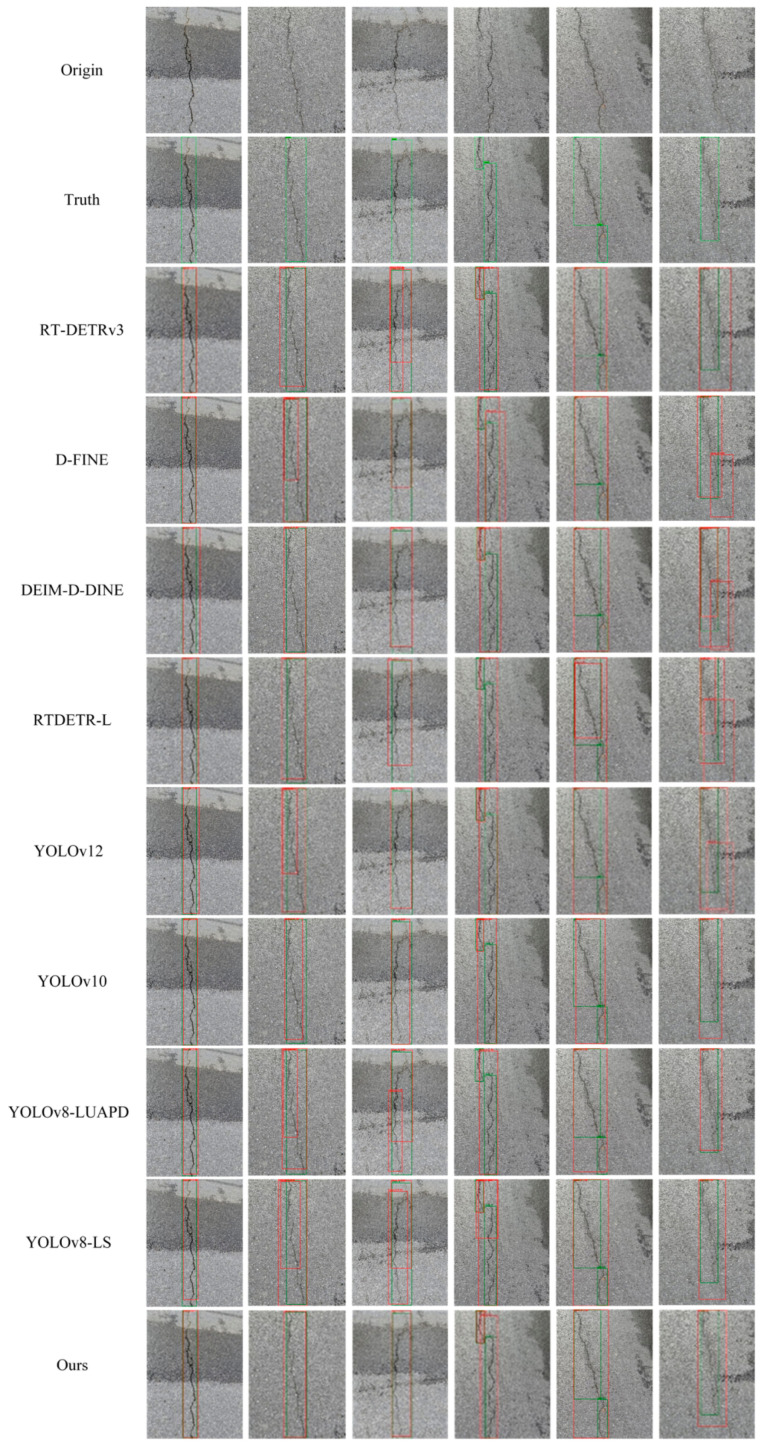
Comparison of detection results of different models in complex scenarios. (Green bounding boxes represent the ground-truth annotations, and red bounding boxes represent the predictions generated by the models, same applies to the following figures.).

Table 6 and Figure 3 present the comparison results between the proposed MCGC-Net and several mainstream object detection models, including both general-purpose detectors and crack-specific crack detection methods. The compared methods include RT-DETRv3-R50 [28], D-FINE-L [29], DEIM-D-FINE-L [30], RTDETR-L [31], YOLOv12L [32], YOLOv10n [33], YOLOv8-LUAPD [34], and YOLOv8s-LS [35].

Among the general-purpose detectors, RT-DETRv3-R50 achieved relatively high Precision (89.31%), indicating strong suppression ability for false-positive predictions. However, its Recall remained relatively limited (76.47%), suggesting insufficient sensitivity when detecting slender and discontinuous crack regions under complex pavement backgrounds. D-FINE-L and RTDETR-L achieved relatively balanced Recall performance, but their Precision and AP50 remained comparatively low in scenes containing pavement textures, shadows, and complex interference regions. YOLOv12L achieved the highest Recall (82.35%) among the compared general-purpose detectors, indicating relatively strong crack coverage capability. However, its lower Precision (76.82%) suggests that the model tends to produce more false-positive responses in complex background regions.

Compared with the general-purpose detectors, the crack-specific lightweight methods YOLOv8-LUAPD and YOLOv8s-LS achieved improved crack localization performance with relatively low computational complexity. YOLOv8-LUAPD achieved 82.58% AP50 with only 2.6M parameters, while maintaining high inference efficiency, demonstrating good lightweight crack detection capability. YOLOv8s-LS further improved AP50 to 83.70% through multi-scale feature optimization while maintaining relatively low computational cost. These results indicate that crack-oriented structural optimization strategies can effectively improve crack detection performance in UAV-based road inspection scenarios.

Compared with all competing methods, the proposed MCGC-Net achieved the best overall detection performance. Precision, Recall, F1-score, and AP50 reached 89.55%, 79.40%, 84.17%, and 86.58%, respectively. In addition, MCGC-Net maintained an inference speed of 45 FPS on the NVIDIA RTX3090 platform, demonstrating acceptable deployment efficiency despite introducing multimodal semantic interaction and nonlinear channel modeling mechanisms.

Although the proposed framework introduces higher computational complexity and parameter counts due to multimodal semantic interaction and nonlinear channel modeling, it demonstrates improved localization stability and feature representation capability under challenging crack scenarios, including slender cracks, discontinuous crack regions, shadow interference, and complex pavement textures. The additional complexity mainly originates from multimodal semantic guidance and nonlinear feature enhancement mechanisms designed to strengthen crack representation capability under complex UAV road inspection conditions.

From a practical deployment perspective, UAV-based road inspection applications usually prioritize crack detection reliability and structural completeness over extreme lightweight deployment. Missing slender crack targets or weak-texture crack regions may directly affect road condition assessment results. Therefore, a moderate increase in parameter count is justified when acceptable inference efficiency can still be maintained while substantially improving crack detection robustness and localization accuracy. The proposed framework is particularly suitable for UAV-based pavement inspection and post-flight road condition assessment scenarios, where detection robustness and structural completeness are generally more critical than extreme real-time performance.

These results indicate that the proposed multimodal semantic guidance and geometry-consistency modeling strategy can effectively improve crack detection robustness under complex UAV-based road inspection conditions while maintaining a favorable balance between detection performance and deployment efficiency.

### 3.6. Generalization Experiments

A generalization test evaluates how well a model performs on unseen data after being trained and generalized from some training data. The above experiments show that the model has some strengths and weaknesses in different environments, such as different data quality, resolutions and light conditions, and its performance in complex background situations.

#### 3.6.1. Generalization Experiment of Peng Dataset

To further evaluate the cross-dataset generalization capability of the proposed method, additional experiments were conducted on the Peng dataset. This dataset contains crack images collected under various complex road conditions, including illumination variation, shadow interference, complex pavement textures, and a large number of fine crack targets. In addition, noticeable differences exist among datasets in terms of imaging devices, scene distributions, and crack morphologies. Therefore, this experiment can more effectively evaluate the adaptability and stability of crack detection models under unseen scenarios.

**Figure 4:**
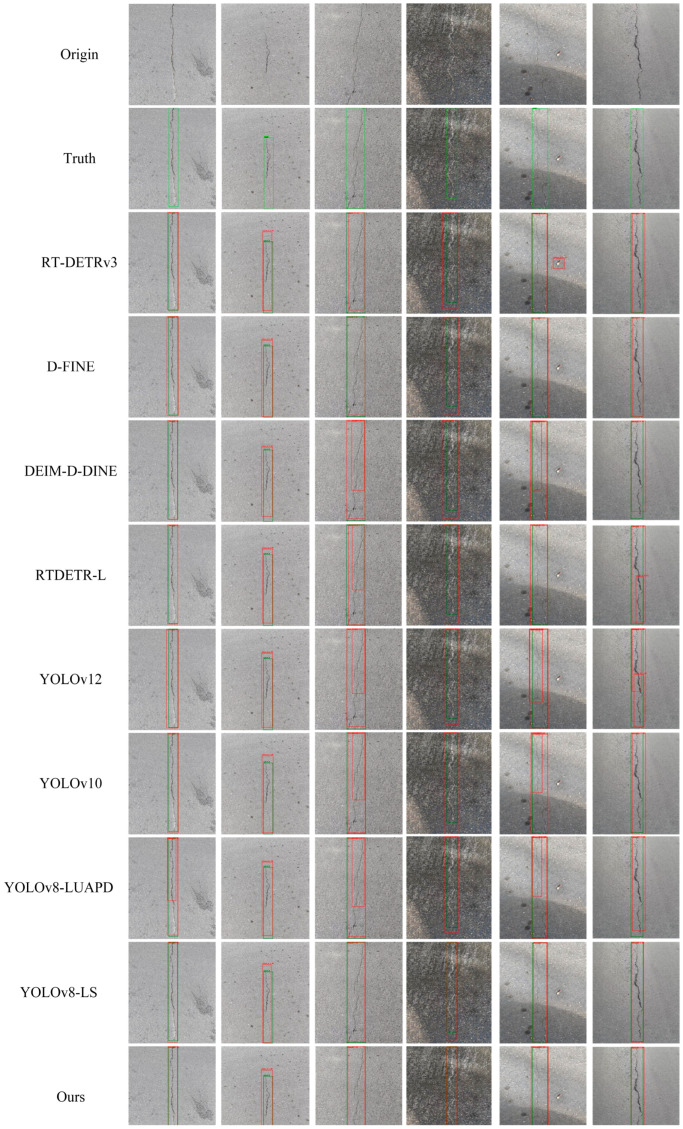
Comparison of generalization performance on the Peng dataset.

As shown in Table 7 and Figure 4, the performance of all compared models decreased to different degrees under the cross-dataset setting, indicating that crack detection across different scenes remains a challenging task. Some models maintained relatively high Recall while suffering from obvious decreases in Precision, suggesting that they tend to produce more false-positive predictions under complex background conditions. In contrast, several other models achieved relatively high Precision but showed limited sensitivity to fine cracks and weak-texture crack regions, resulting in lower Recall values. These results indicate that detection methods relying mainly on visual feature extraction are still easily affected by background texture variation, illumination changes, and crack structure differences under cross-dataset conditions.

Compared with the competing methods, the proposed MCGC-Net maintained relatively stable overall performance on the Peng dataset. Specifically, Precision, Recall, F1-score, and AP50 reached 84.91%, 77.24%, 81.89%, and 80.89%, respectively. In particular, the proposed method still achieved clear advantages in Precision and AP50 compared with other detection models. These results indicate that the proposed method can effectively suppress false-positive responses under complex backgrounds while maintaining relatively stable crack localization capability.

Further analysis shows that RT-DETRv3 achieved relatively high Precision but lower Recall, indicating that the model tends to adopt a more conservative prediction strategy under cross-dataset conditions. Although this strategy helps reduce false-positive predictions, it also leads to missed detections in fine-crack and weak-texture crack regions. YOLOv12 achieved relatively high Recall, indicating strong crack coverage capability. However, its lower Precision suggests that the model is more easily affected by shadows, pavement textures, and noise regions under complex background conditions. D-FINE and RTDETR-L achieved relatively balanced overall performance, but they still showed limitations in simultaneously suppressing false positives and maintaining fine-grained crack localization ability under challenging scenes.

Compared with the general-purpose detectors, the crack-specific lightweight models YOLOv8-LUAPD and YOLOv8s-LS demonstrated relatively better crack localization capability under the cross-dataset setting. Among them, YOLOv8-LUAPD achieved relatively stable detection results with a small parameter scale, while YOLOv8s-LS further improved adaptability to cracks at different scales through multi-scale optimization strategies. These results suggest that structural optimization designed for crack characteristics can improve detection stability in complex UAV-based road inspection scenarios to a certain extent.

Overall, the proposed method maintained relatively stable detection performance under the cross-dataset setting, indicating that the multimodal semantic guidance and geometry-consistency modeling strategy contribute positively to crack feature representation under complex conditions. In particular, the proposed method still maintained relatively stable crack recognition capability under challenging scenarios involving slender cracks, discontinuous crack regions, and complex background interference, demonstrating relatively good cross-scene adaptability and detection robustness.

#### 3.6.2. Generalization Experiment of RDD2022 Dataset

To further evaluate the generalization capability of the proposed method under complex cross-scene conditions, additional experiments were conducted on the RDD2022 dataset. This dataset contains crack images collected from different countries and road environments, resulting in large variations in scene distribution and background conditions.

**Figure 5:**
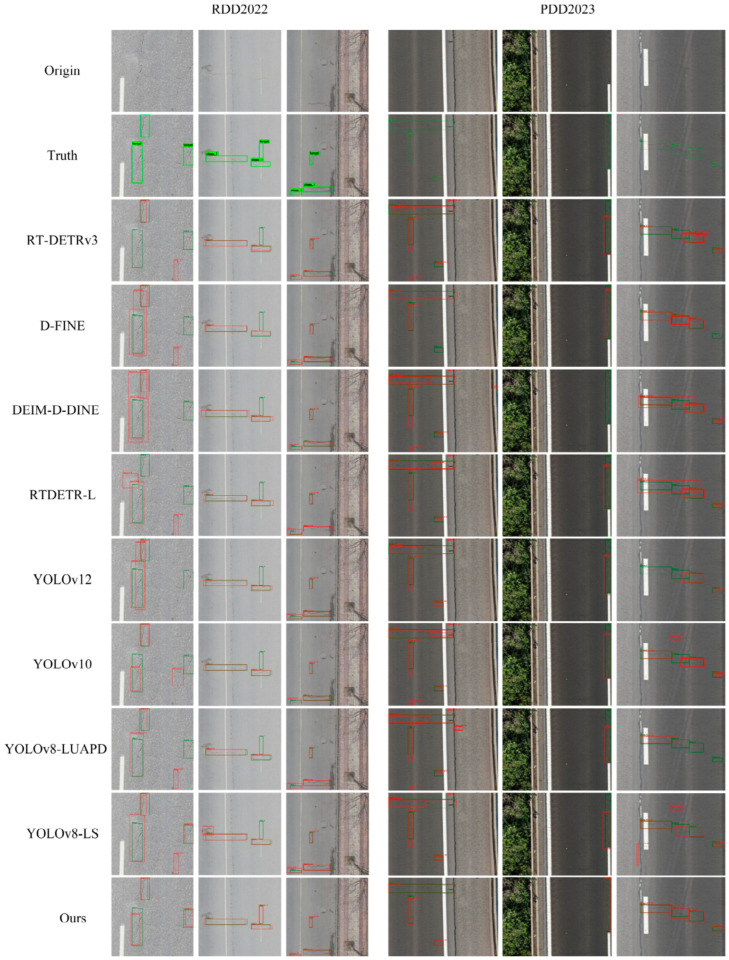
Comparison of generalization performance on the RDD2022 and PDD2023 datasets.

As shown in Table 7 and Figure 5, all compared models experienced performance degradation on the RDD2022 dataset, indicating that cross-dataset crack detection remains a challenging task. Compared with the competing methods, the proposed MCGC-Net achieved the best overall performance, with Precision, Recall, F1-score, and AP50 reaching 61.94%, 72.49%, 66.80%, and 60.31%, respectively. Crack-specific lightweight models such as YOLOv8-LUAPD and YOLOv8s-LS achieved more stable detection performance than general-purpose detectors, but their overall results still remained lower than those of the proposed method. These results indicate that the proposed multimodal semantic guidance and geometry-consistency modeling strategy can effectively improve crack feature representation and detection robustness under complex cross-scene conditions.

Although MCGC-Net achieved the best overall performance, noticeable performance degradation can still be observed compared with the private dataset. One possible reason is domain shift caused by differences in crack morphology, pavement texture, image resolution, illumination conditions, and acquisition environments across datasets. In addition, the proposed framework is mainly optimized on the private UAV crack dataset, which may limit cross-domain feature generalization capability when facing significantly different scene distributions.

Future work will further investigate domain adaptation strategies, domain-oriented data augmentation methods, and lightweight cross-domain fine-tuning approaches using a small number of target-domain samples to improve model robustness under diverse road inspection scenarios.

#### 3.6.3. Generalization Experiment of PDD2023 Dataset

To further evaluate the adaptability of the proposed method in UAV-based road inspection scenarios, additional experiments were conducted on the PDD2023 dataset. This dataset contains complex pavement textures, weak crack boundaries, and various background interference conditions, making crack detection more challenging.

As shown in Table 7 and Figure 5, all compared models showed noticeable performance degradation on the PDD2023 dataset. Among them, the proposed MCGC-Net still achieved the best overall detection performance, with Precision, Recall, F1-score, and AP50 reaching 67.98%, 61.83%, 64.76%, and 49.24%, respectively. Compared with the competing methods, the proposed method maintained relatively stable crack localization capability and background suppression ability under complex UAV scenes. These results indicate that the proposed multimodal semantic guidance and geometry-consistency modeling strategy provides advantages for fine-grained crack detection under complex road inspection conditions.

Compared with the private dataset, the larger performance drop observed on PDD2023 indicates that weak crack boundaries, complex pavement textures, and diverse background interference conditions introduce additional challenges for cross-dataset crack detection. Moreover, limited training diversity and potential overfitting to private dataset characteristics may further affect model adaptability under significantly different visual distributions.

Future work will focus on introducing more diverse training samples, improving cross-domain representation learning capability, and incorporating domain adaptation mechanisms to further improve generalization performance under complex practical deployment scenarios.

## 4. Discussion

The proposed MCGC-Net takes UAV road images and crack description text as inputs. During preprocessing, all road images are resized to a unified resolution to ensure compatibility with the network structure. Subsequently, the image data and corresponding crack description text are jointly integrated to construct a visual-semantic crack detection framework. The extracted features are then processed through the Multimodal Contrastive Semantic Gating Module (MCSG), Crack-Aware Slenderness Loss (CASL), and KAN-based nonlinear channel attention mechanism. Finally, the network outputs the crack detection results and visualizes the predicted crack regions. Through the integration of semantic guidance and crack-oriented feature modeling, the proposed framework can improve crack localization capability and overall detection stability under complex UAV road inspection conditions.

Experimental results demonstrate that MCGC-Net achieves superior crack detection performance compared with several competing methods. However, the proposed model still has several limitations:

(1) The proposed framework introduces multimodal semantic interaction, nonlinear channel modeling, and geometry-consistency constraints during crack detection. Although these designs improve crack feature representation capability and detection robustness, they also increase the overall parameter scale and computational complexity of the network. Compared with lightweight crack detection models, the proposed method still has limitations in deployment on edge devices, low-power UAV platforms, and real-time inspection scenarios. Therefore, the real-time inference performance of the current framework still requires further improvement.

(2) Although MCGC-Net performs well for most crack scenarios, the model still shows limited detection capability in several challenging cases, including large-area damage regions, heavily interconnected crack structures, and low-contrast crack regions. This limitation may be related to the complexity of crack morphologies, variations in pavement materials, and the relatively limited number of such challenging samples in the current training dataset.

(3) At present, the training dataset of MCGC-Net mainly consists of crack images collected from specific road scenes. The current dataset contains only 800 images, including 600 training images, 100 validation images, and 100 testing images. Although the dataset covers multiple pavement types and crack conditions, the relatively limited dataset scale may still constrain feature generalization capability under significantly different deployment environments. Samples from other pavement structures, road materials, and environmental conditions are still relatively limited. This dataset bias may affect the cross-scene generalization capability of the proposed model. When applied to unseen environments, such as bridge decks, airport pavements, or roads with different pavement structures, the detection performance of the model may decrease to some extent.

To alleviate the small-sample limitation, conventional data augmentation strategies were employed during model training to improve data diversity and training stability. In addition, pretrained visual feature extraction networks were adopted to improve feature learning capability under limited training samples. Furthermore, the proposed template-based crack description generation strategy enables semantic knowledge reuse across samples, helping strengthen multimodal representation capability under relatively limited data conditions.

(4) The current framework mainly relies on conventional preprocessing operations, including image denoising, normalization, and data augmentation, to improve training stability and feature consistency. However, under certain complex environmental conditions, such as strong illumination, shadow interference, rainwater disturbance, and low-light scenes, the preprocessing strategy may still be insufficient to completely suppress environmental noise. As a result, the detection performance of the model may still be affected in these challenging scenarios.

Future work can further improve the robustness and practical application capability of the proposed method from several aspects. (1) Future studies can further focus on lightweight network design and model compression strategies, including pruning, quantization, and knowledge distillation, to reduce model parameters and computational cost while maintaining detection performance. (2) More environmental information, such as illumination conditions, weather conditions, and pavement material characteristics, can be introduced during training to improve the adaptability of the model under complex road inspection scenarios. (3) Future work can further expand the training dataset by introducing more road environments, pavement types, and crack categories, thereby improving the cross-scene generalization capability of the model. (4) Future research can further optimize the image preprocessing pipeline, especially in denoising, normalization, and data augmentation stages, to improve detection stability under different crack scales and environmental conditions. (5) Future work can further improve the real-time inference capability of the proposed framework to better support online road inspection and intelligent maintenance applications in practical UAV-based road monitoring systems.

Overall, this study develops an effective UAV-based crack detection framework for accurate crack localization and recognition in complex road environments. The proposed method can provide useful support for road maintenance management, early pavement damage assessment, and transportation infrastructure safety monitoring.

## 5. Conclusions

Through this work, we also provide solutions to several issues in UAV road crack detection, especially for three practical issues. First, slender cracks could not be detected accurately. Second, complicated background patterns always introduced false positive results. Third, cracks exhibited nonlinear shapes which also made accurate detection hard. In our research, we constructed a UAV road crack dataset. We used a multirotor UAV with a high-resolution RGB camera to collect the images. In total, we have 800 images in the dataset. The images cover different pavement types, including asphalt and concrete, and contain various scales of cracks with different light and background conditions. We split the dataset into training images (600), validation images (100) and testing images (100). This data split provides a good and uniform basis for training models and performance evaluation of models.

Based on the above dataset, we built a crack detection system containing three parts: MCSG, CASL and KAN-CA. MCSG is aimed at improving a model’s ability to acquire crack-related semantic information and is more effective in complicated back-end environments. Adaptive spatial modeling and multi-scale feature modeling are controlled in this part. We also added a text-sense cue of the same type for feature learning and eliminated semantic errors at this time to help the model further identify true cracks from other non-interest background patterns in subsequent processing stages. We primarily considered cracks with thin walls and narrow bases. At this time, we added a slenderness consistency constraint to the objective of optimizing long crack targeting, thus making the regression process less susceptible to deviation, as well as proving that traditional IoU-type loss cannot be applied to such types of target problems. KAN-Ca adds another function to enhance feature representation through a non-computable channel mapping operation. This design can better represent crack deflection, extension and local changes in a structure’s micro-crack.

The experiment has demonstrated its effectiveness in realizing the above scheme. Comparatively speaking, our approach had a higher precision (89.55%), mean average-precision at k = 50 point (86.58%) and recall rate (F1-score) of 20% (84.17%). Recall reached 79.4 per cent, a result also surpassing some competitive systems’ scores. It can be inferred that this improvement was not due to detecting more crack areas. Therefore, the result was more suitable for maintaining this degree of reasonable balance between cracks covered, false-positives suppressed and overall detection accuracy. The ablation tests also confirmed that these three parts cooperated reasonably well with each other. MCSG was more effectively improved among these groups. The CASL was needed to enhance the localization of slender cracks as well. KAN-CA also improved the crack-fitting performance of this model through additional complexity in shape representation. MCSG, CASL, and KAN-CA can be combined to achieve the highest generalization ability of this system.

In general, the above approach demonstrates excellent performance in UAV-assisted road-crack detection. It can achieve the required accuracy and stability. Therefore, it has certain applications and prospects. In future work, we will also add more modalities of information beyond just RGB images. In addition, we may reduce the size of the model further in the future through decompression work to enhance the generalization performance of this approach for real-world road-photo-based identification tasks and edge-environment applications.

## Figures and Tables

**Table 1:** Hardware and software parameters.

Hardware environment	CPU	AMD Ryzen 7 5800H
	GPU	NVIDIA GeForce RTX 3090
	RAM	64 GB
	Video Memory	24 GB
Software environment	OS	Windows 10 × 64
	CUDA Toolkit	V10.2
	CUDNN	V8.2.1
	Python	3.8

**Table 2:** Experimental comparison of MCSG effectiveness.

Method	Precision	Recall	F1-Score	AP_50_
Baseline (3 × 3 conv)	81.25%	74.73%	77.85%	80.65%
DCNv2	82.68%	75.94%	79.17%	78.92%
DyConv	82.45%	75.62%	78.89%	78.64%
MCSG (without contrastive gating)	83.67%	77.79%	80.62%	82.70%
MCSG (without multi-scale aggregation)	83.12%	76.58%	79.72%	82.14%
Ours	84.31%	76.97%	80.47%	83.72%

**Table 3:** Experimental comparison of CASL effectiveness.

Method	Precision	Recall	F1-Score	AP_50_
CIoU Loss	80.29%	74.15%	77.36%	78.63%
EIoU Loss	81.68%	75.12%	78.26%	79.14%
Focal-EIoU Loss	82.41%	75.92%	79.03%	78.51%
CSLS loss	83.12%	76.45%	79.65%	78.62%
CSLS loss (3 × 3)	83.61%	76.24%	79.76%	81.42%
CSLS loss (5 × 5)	82.89%	76.18%	79.39%	80.34%
Ours	84.15%	76.58%	80.19%	82.13%

**Table 4:** Experimental comparison of KAN-CA effectiveness.

Method	Flops (G)	Parm (M)	Precision	Recall	F1-Score	AP_50_
Our model base	21.7	8.9	81.25%	74.73%	77.85%	80.65%
SE-Net	25.9	10.6	82.15%	75.38%	78.62%	81.42%
ECA	23.1	11.8	82.47%	75.61%	78.89%	81.68%
DSConv	27.6	10.4	82.64%	76.72%	79.56%	82.75%
KAN-CA (grid = 3, order = 2)	47.2	20.5	83.28%	76.45%	79.72%	82.67%
KAN-CA (grid = 7, order = 4)	49.5	22.3	82.95%	76.89%	79.81%	82.45%
OUrs	45.4	19.1	83.36%	77.45%	80.30%	83.34%

**Table 5:** Results of ablation experiment.

Group	Multimodal Input	MCSG	CASL	KAN-CA	Precision	Recall	F1-Score	AP_50_
1	-	-	-	-	81.25%	74.73%	77.85%	80.65%
2	√				82.55%	75.41%	78.37%	81.69%
3	√	√			84.91%	77.24%	80.89%	84.21%
4	√		√		84.31%	76.82%	80.39%	82.13%
5	√			√	83.67%	77.79%	80.89%	83.70%
6	√	√	√		88.81%	78.43%	83.30%	85.18%
7	√	√		√	86.31%	77.12%	81.46%	85.00%
8	√		√	√	86.73%	78.68%	82.51%	85.71%
9	√	√	√	√	89.55%	79.40%	84.17%	86.58%

*√ indicates the presence of the corresponding module, while “-” indicates its absence*.

**Table 6:** Comparative experiment results for different networks.

Network	Flops (G)	Parm (M)	FPS	Precision	Recall	F1-Score	AP_50_
RT-DETRv3-R50	136	42	27	89.31%	76.47%	82.39%	72.94%
D-FINE-L	91	31	50	69.31%	75.73%	74.16%	74.87%
DEIM-D-FINE-L	95	34	39	79.59%	76.747%	78.14%	73.59%
RTDETR-L	125	47	28	71.51%	77.12%	74.21%	72.97%
YOLOv12L	88.9	26.4	61	76.82%	82.35%	79.49%	79.76%
YOLOv10	8.2	2.7	223	81.25%	74.73%	77.85%	80.65%
YOLOv8-LUAPD	20.1	2.6	170	86.83%	78.39%	81.47%	82.58%
YOLOv8s-LS	13.4	6	165	87.62%	77.65%	82.13%	83.70%
Ours	95.7	42.8	45	89.55%	79.40%	84.17%	86.58%

**Table 7:** Experimental results of generalization.

Group	Dataset	Model	Precision	Recall	F1-Score	mAP_50_
	Peng	RT-DETRv3	82.45%	74.23%	80.34%	71.56%
		D-FINE	67.82%	77.15%	72.56%	73.24%
		DEIM-D-FINE	77.68%	75.42%	76.48%	72.18%
		RTDETR-L	69.85%	75.68%	72.67%	71.45%
a		YOLOv12	75.34%	80.67%	77.89%	78.12%
		YOLOv10	78.75%	73.45%	74.84%	74.41%
		YOLOv8-LUAPD	81.56%	74.60%	78.96%	77.52%
		YOLOv8s-LS	82.49%	75.91%	77.73%	78.35%
		Ours	84.91%	77.24%	81.89%	80.89%
	RDD2022	RT-DETRv3	54.52%	60.65%	57.42%	54.38%
		D-FINE	53.15%	59.42%	56.11%	52.45%
		DEIM-D-FINE	53.57%	59.36%	56.31%	53.62%
		RTDETR-L	54.79%	60.59%	57.54%	55.71%
b		YOLOv12	56.32%	62.71%	59.34%	55.97%
		YOLOv10	55.39%	61.66%	58.36%	54.87%
		YOLOv8-LUAPD	57.51%	69.68%	63.01%	58.66%
		YOLOv8s-LS	58.78%	70.38%	64.06%	57.93%
		Ours	61.94%	72.49%	66.80%	60.31%
	PDD2023	RT-DETRv3	59.64%	53.87%	56.61%	44.37%
		D-FINE	61.48%	50.45%	55.42%	43.53%
		DEIM-D-FINE	58.78%	50.85%	54.53%	45.18%
		RTDETR-L	57.82%	51.84%	54.67%	44.92%
c		YOLOv12	61.16%	55.14%	57.99%	44.87%
		YOLOv10	59.66%	54.45%	56.94%	45.81%
		YOLOv8-LUAPD	65.11%	59.55%	62.21%	46.51%
		YOLOv8s-LS	65.61%	58.81%	62.02%	45.98%
		Ours	67.98%	61.83%	64.76%	49.24%

## Data Availability

The datasets used in this study consist of two parts. One part is a self-constructed dataset developed by the authors and used for model training and evaluation. The other part is a publicly available dataset previously released by our research group. The public dataset can be accessed through the corresponding published work or related platforms. The self-constructed dataset is not publicly available at this stage due to ongoing research but can be obtained from the corresponding author upon reasonable request.
